# The Alzheimer’s Prevention Initiative Composite Cognitive Test: a practical measure for tracking cognitive decline in preclinical Alzheimer’s disease

**DOI:** 10.1186/s13195-020-00633-2

**Published:** 2020-05-27

**Authors:** Jessica B. Langbaum, Noel N. Ellison, Angelika Caputo, Ronald G. Thomas, Carolyn Langlois, Marie-Emmanuelle Riviere, Ana Graf, Cristina Lopez Lopez, Eric M. Reiman, Pierre N. Tariot, Suzanne B. Hendrix

**Affiliations:** 1 Banner Alzheimer’s Institute, 901 E. Willetta Street, Phoenix, AZ USA; 2 Pentara Corporation, 2261 E. 3300 S. Suite 200, Salt Lake City, UT USA; 3 Novartis Pharma AG, Forum 1, Novartis Campus, CH-4056 Basel, Switzerland; 4 University of California San Diego School of Medicine, 9500 Gilman Dr, La Jolla, CA USA

**Keywords:** Preclinical Alzheimer’s disease, Cognition, Composite cognitive endpoint, Clinical trials

## Abstract

**Background:**

There is growing interest in identifying sensitive composite cognitive tests to serve as primary endpoints in preclinical Alzheimer’s disease (AD) treatment trials. We reported previously a composite cognitive test score sensitive to tracking preclinical AD decline up to 5 years prior to clinical diagnosis. Here we expand upon and refine this work, empirically deriving a composite cognitive test score sensitive to tracking preclinical AD decline up to 11 years prior to diagnosis and suitable for use as a primary endpoint in a preclinical AD trial.

**Methods:**

This study used a longitudinal approach to maximize sensitivity to tracking progressive cognitive decline in people who progressed to the clinical stages of AD (*n* = 868) compared to those who remained cognitively unimpaired during the same time period (*n* = 989), thereby correcting for normal aging and practice effects. Specifically, we developed the Alzheimer’s Prevention Initiative Preclinical Composite Cognitive test (APCC) to measure very early longitudinal cognitive decline in older adults with preclinical AD. Data from three cohorts from Rush University were analyzed using a partial least squares (PLS) regression model to identify optimal composites within different time periods prior to diagnosis, up to 11 years prior to diagnosis. The mean-to-standard deviation ratio (MSDRs) is an indicator of sensitivity to change and was used to inform the final calculation of the composite score.

**Results:**

The optimal composite, the APCC, is calculated: 0.26*Symbol Digit Modalities + 2.24*MMSE Orientation to Time + 2.14*MMSE Orientation to Place + 0.53*Logical Memory Delayed Recall + 1.36* Word List-Delayed Recall + 0.68*Judgment of Line Orientation + 1.39*Raven’s Progressive Matrices Matrices (subset of 9 items from A and B). The MSDR of the APCC in a population of preclinical AD individuals who eventually progress to cognitive impairment, compared to those who remained cognitively unimpaired during the same time period, was − 1.10 over 1 year.

**Conclusions:**

The APCC is an empirically derived composite cognitive test score with high face validity that is sensitive to preclinical AD decline up to 11 years prior to diagnosis of the clinical stages of AD. The components of the APCC are supported by theoretical understanding of cognitive decline that occurs during preclinical AD. The APCC was used as a primary outcome in the API Generation Program trials.

## Introduction

Alzheimer’s disease (AD) is the most common cause of dementia. Findings from numerous studies suggest that the pathophysiological process of AD begins years, if not decades, before clinical symptoms of AD are evident [1–3]. We hypothesize that, to have their greatest benefit, AD-modifying treatments may need to be started in the preclinical AD phase, before the onset of cognitive impairment or other clinical symptoms, to have their most profound effect [4]. Several such preclinical AD trials are underway, with many more in various stages of planning [5].

Most existing cognitive assessment scales used as outcome measures in AD trials have been designed primarily for studies in individuals with mild cognitive impairment (MCI) or dementia. These assessments are not suitable endpoints for use in preclinical AD trials due to their inability to detect or track subtle cognitive change during the preclinical stages of the disease [6, 7]. Likewise, the sensitivity of these scales for early-stage, preclinical AD has been questioned and the need for better-targeted measures emphasized [8]. As a result, there is growing interest in use of composite cognitive endpoints as primary endpoints in preclinical AD trials [9–12] and recognition of the importance of selecting the combination of tests and/or items most sensitive to detect changes in disease progression and a drug treatment effect [13].

We reported previously a strategy to empirically determine the combination of cognitive assessments most sensitive to track cognitive change in preclinical AD while controlling for normal aging and practice effects, creating a late-onset AD composite cognitive test score [12]. In this initial work, the mean-to-standard deviation ratio (MSDR) of the change score was employed as the measure of sensitivity to preclinical AD decline over time. A computationally-intense “brute force” approach was used, which evaluated the responsiveness of all the possible combinations of two to seven cognitive test items from a larger test battery. Data were available for participants up to 5 years prior to the diagnosis of MCI or dementia, as well as data from individuals who remained cognitively unimpaired during the same time period to control for aging and practice effects. The composite consisted of Category Fluency (fruits and vegetables), Boston Naming Test (15 items), Logical Memory Delayed Recall, East Boston Naming Test Immediate Recall, Raven’s Progressive Matrices Subset, Symbol Digit Modalities, and MMSE Orientation to Time [12].

In preparation for the Alzheimer’s Prevention Initiative (API) Generation Program trials [14], we needed to ensure the primary composite endpoint was optimized for tracking preclinical cognitive decline. In the present study, we report our findings to extend and validate our initial work [12] by using a partial least squares (PLS) modeling approach using data up to 11 years prior to the diagnosis of MCI or dementia. The PLS modeling approach is a more direct way to achieve the results compared to the “brute force” approach employed originally, allowing for the consideration of the correlations between individual test items rather than assessing items individually. The PLS model, with time as the outcome variable, identified the optimal weighted linear combination of predictors for maximizing both consistency and responsiveness to disease progression over time.

## Methods

### Participants

Data from the Rush Alzheimer’s Disease Center’s Religious Orders Study (ROS), Memory and Aging Project (MAP), and the Minority Aging Research Study (MARS) were downloaded in December 2012. Enrollment criteria for each of these studies have been described elsewhere [15–17]. Briefly, participants were cognitively unimpaired older adults who underwent annual clinical and neuropsychological evaluations. Longitudinal data from participants who progressed to either MCI (all cause) or AD dementia (possible or probable) and who had at least one follow-up visit were used to select items to be included in the composite. Data from participants who remained cognitively unimpaired during the same time period (7-year buffer from diagnosis) were used to control for aging and practice effects but were not directly included in the derivation of the composite score. All studies were approved by the Rush University Medical Center Institutional Review Board, and informed consent was obtained from each participant.

### Cognitive assessments

All three Rush studies used structured annual clinical and neurological evaluations as well as neurocognitive testing by trained clinicians [18, 19]. The cognitive batteries administered to participants in each study are shown in Table 1 [12].

**Table 1:** Cognitive assessments in the Rush Alzheimer’s Disease Center cohort studies

Cognitive assessment	Domain	ROS	MAP	MARS
Boston Naming Test (15 items)	Language/semantic memory	X	X	X
Category fluency—animals	Language/semantic memory	X	X	X
Category fluency—fruits/vegetables	Language/semantic memory	X	X	X
CERAD Word List Recall (Immediate)	Episodic memory	X	X	X
CERAD Word List Memory (Delayed Recall)	Episodic memory	X	X	X
CERAT Word List Recognition	Episodic memory	X	X	X
Complex Ideational Material	Auditory comprehension	X	X	X
Digit Ordering	Working memory	X	X	X
Digit Span—Forward	Working memory	X	X	X
Digit Span—Backward	Working memory	X	X	X
East Boston Naming Test, Immediate Recall (Memory I)	Episodic memory	X	X	X
East Boston Naming Test, Delayed Recall (Memory II)	Episodic memory	X	X	X
Judgment of Line Orientation	Visuospatial	X	X	X
Logical Memory Ia (Immediate)	Episodic memory	X	X	X
Logical Memory IIa (Delayed)	Episodic memory	X	X	X
Mini-Mental State Examination (MMSE)—Total	General/global cognition	X	X	X
MMSE—Orientation to Time	Orientation	X	X	X
MMSE—Orientation to Place	Orientation	X	X	X
MMSE—Registration	Working memory	X	X	X
MMSE—Attention and Concentration	Attention and concentration	X	X	X
MMSE—Recall	Episodic Memory	X	X	X
MMSE—Language	Language	X	X	X
National Adult Reading Test (10 items)	General/global cognition	X	X	
Number Comparison Test	Perceptual speed	X	X	X
Ravens Progressive Matrices (16 items)	Visuospatial/working memory	X	X	X
Ravens Progressive Matrices Subset (9 items from A and B)	Visuospatial/working memory	X	X	X
Symbol Digit Modalities	Perceptual Speed	X	X	X
Wide Range Achievement Test (15 items)	General/global cognition			

Abbreviations: *ROS* Religious Orders Study, *MAP* Memory and Aging Project, *MARS* Minority Aging Research Study (MARS)

### Diagnostic classifications

Participants were classified diagnostically according to recommendations from the joint working group of the National Institute of Neurologic and Communicative Disorders and Stroke and the Alzheimer’s Disease and Related Disorders Association [20]. When possible, dementia diagnoses were validated pathologically [20–23] and supported by evidence from medical history (obtained by structured interview) and current cognitive impairment (obtained by neuropsychological testing). Dementia was attributed to AD or other causes based on medical chart review, structured clinical interview, and examination by a clinician with expertise in dementia evaluations. A diagnosis of MCI was given when participants exhibited cognitive impairment, as determined by neuropsychological testing, but did not meet criteria for dementia, as determined by the clinician [21, 24, 25].

### Data preparation

The primary population of interest for analysis was the individuals who were initially cognitively unimpaired but were eventually diagnosed with either AD (dementia due to AD, or AD and other diagnoses) or MCI (all causes), designated as “progressors.” The longitudinal data for these participants up to 11 years prior to a diagnosis were included in the analysis. Item scores were standardized to a range of 0.0 to 1.0 as shown in the following equation:

$$ \mathsf{Standardized}\ \mathsf{score}=\left(\mathsf{Original}\ \mathsf{score}-\mathsf{Minimum}\ \mathsf{possible}\ \mathsf{score}\right)/\left(\mathsf{Maximum}\ \mathsf{score}\ \mathsf{possible}-\mathsf{Minimum}\ \mathsf{score}\ \mathsf{possible}\right) $$

After this standardization, all items were comparable on a 0 to 1 scale with 0 representing the worst score and 1 representing the best cognitive performance. These standardized scores were used for all analyses. *Z*-scores were not used since the objective was to take advantage of the full range of the scale, rather than have scores relative to a specific population.

### Cognitive aging and practice effects adjustment

A cognitive aging adjustment was made to correct for differences in decline due to age effects and practice effects. This adjustment was made by analyzing data from participants who did not progress to MCI or dementia and had at least 7 years without a diagnosis beyond the time included in the analysis. A multiple regression was used with age as the first predictor, visit year of the assessment as the second predictor, and the change in each item as the outcome variable. Quadratic effects for age and visit year were also included, as was the interaction between age and visit. The adjusted score for each progressor was then calculated by subtracting the predicted change score for a cognitively unimpaired person of the same age at each visit. These adjusted values for progressors were then used in the derivation of the composite score.

### Determining selection of the APCC scales

The approach to optimizing a composite measure was based on the goal of identifying the combination of items and associated weights ideally suited for measuring disease-related cognitive changes. The mean-to-standard deviation ratio (MSDR) was used as a metric to assess composite performance, and a partial least squares (PLS) regression model, with time as the outcome variable, was used to directly identify the linear combination of items (items and weights) for maximizing the responsiveness to disease progression over time. A theoretical evaluation was performed to eliminate any non-contributing items and to ensure that relevant cognitive domains were well represented with the included items.

### Mean-to-standard deviation ratio metric for sensitivity

The MSDR of the change scores over time was used as a performance metric after correcting for normal aging and practice effects because it corresponds directly to assessing the power of a composite score in detecting treatment effects in a clinical trial for a disease-modifying treatment. This correspondence relies on the assumption that a treatment effect would be proportional to the cognitive decline rate of the group that progresses to AD relative to the group that does not. In other words, a treatment for preclinical AD would be expected to affect only AD-related progression, and not to slow normal aging. A lower standard deviation would then make that change easier to detect in a clinical trial.

MSDR was calculated using the following equation:

$$ \mathsf{MSDR}=\mathsf{Mean}\ \mathsf{annual}\ \mathsf{change}\ \mathsf{from}\ \mathsf{baseline}/\mathsf{SD}\ \mathsf{of}\ \mathsf{annual}\ \mathsf{change}\ \mathsf{from}\ \mathsf{baseline} $$

The MSDR criterion was used as the primary metric for assessing performance of item combinations. The neuropsychological tests that were included in this analysis are considered valid measurements of cognitive performance, and the MSDR allows assessment of the external validity related to progressive symptoms relative to a normal population. Reliability is an inherent part of the MSDR calculation since less reliable outcomes have larger variability over time resulting in lower MSDRs [26].

### PLS methodology

A PLS modeling approach was implemented to identify the optimal combination of items and associated weights for sensitively measuring change over time in progressors to MCI or AD dementia. Although the items/tests are implicitly weighted due to their unique scale properties, we could not assume these weights would be the optimal choice. If the implicit weights are close to optimal, the PLS model may derive weights that are nearly identical to the implicit weights. A PLS model was selected because of its ability to integrate the modeling of progression with dimension reduction methods that account for correlations between individual items. The PLS model, with time as the outcome variable, identified the best weighted linear combination of predictors for maximizing both consistency and responsiveness to disease progression over time. All analyses were conducted using SAS, version 9.4 (SAS Institute Inc.).

The original predictive method for PLS regression was proposed by Wold [27]. The PLS procedure extracts one factor at a time, refitting the next model to the residuals of the previous model. The number of factors extracted from the PLS procedure was based on cross-validation using van der Voet’s [28] randomization-based model comparison test based on Hoteling’s *t* statistic, and 1000 randomizations using a leave-one-out cross-validation approach and an alpha level of 0.10 indicating a factor should be kept in the model. The weights for each item in the model were summed across the extracted factors.

Variable Importance for Projection (VIP) is a statistic summarizing the contribution each variable makes to the model. If a predictive variable has an average influence on the model, its VIP value will be 0.5. Wold [29] considers a value less than 0.8 to be “small” for the VIP. From this follows a recommended practice of setting a cut-off value of 0.8 to separate out the most important predictive variables from the least important. The VIP criterion of 0.8 was used for item selection and the weights identified by the PLS model were used for calculating potential composite scores. The MSDR of each composite score within a patient population was used to assess the performance of the composite for measuring progression over time.

### Step 1—accounting for differences in time until diagnosis

Analyses were conducted on three different time periods prior to the diagnosis of MCI or AD dementia. Because the pattern of progression differs during different stages of disease, the process for defining the APCC involved balancing the optimized combination across different time periods prior to a diagnosis of MCI or AD dementia. The population included in the clinical trial is a mix of participants who will progress to the clinical stages of AD during the trial and participants who will not be diagnosed but will still experience preclinical progression and may be within 5–6 years of a diagnosis at the end of a 5-year study. Progression during the year of diagnosis affects more items and is much faster than progression during the 5 years prior to diagnosis (− 5 to − 1) and even fewer items are changing and at a very slow rate of progression in the 6 to 11 years prior to diagnosis. Inclusion of data from periods of very fast progression, such as the year of diagnosis, resulted in an over-weighting of items that were responsive to change in that period, particularly if only a few earlier years were included in the model. To reflect this mixed population of very early, early, and just diagnosed participants, separate PLS models were run for 3 time periods: 10 years prior to diagnosis and year of diagnosis (− 10 to 0 years, to measure the impact of including the year of diagnosis), − 11 years prior to year of diagnosis (− 5 to − 1 years, to measure the pre-diagnosis changes), and 5 years prior to year of diagnosis (− 5 to − 1 years, to assess the increase in rate of change near diagnosis, without including the highly influential year of diagnosis). Although the 10-year and 11-year time period overlap almost entirely, only the 10-year time period includes the year of diagnosis, during which large changes occur.

The composite score was selected to achieve consistency of items across the time periods since the population of the study would include a cross-section of individuals from different time periods prior to a diagnosis. The items for the APCC were selected as the specific set of items that could best cover the stages of preclinical AD. Although some clinical changes could be evident prior to the 11-year time frame, not enough participants were available with more than 11 years of data prior to a diagnosis.

### Step 2—theoretical considerations for selection of the composite

Domains of cognition that are relevant to the decline in this preclinical stage were identified based on the PLS results after the removal of items that weakened the combination (decreased the MSDR). Important domains should ideally be measured by multiple, sensitive items to improve estimate stability. However, if only one item in a domain was identified as a sensitive measure of progression, that item/domain was still included.

### Step 3—assessing individual item contribution to the combination

Each component was then removed one at a time from this combination to assess the impact on the performance of the combination as measured by the estimated MSDR over each time period. Items that resulted in an improved MSDR when the item was removed were excluded from the final composite score.

### Step 4—create final composite with weights for raw scores

Following identification of the final test items using the 3 steps above, an additional PLS analysis was performed using non-standardized values to identify the optimal weighting for the raw item scores on their original scales. The test score was then standardized to a 0–100 range, by dividing each original weighted item maximum by the original maximum sum and then multiplying by 100, with a higher score corresponding to a better disease condition.

## Results

### Participants

Our analysis included 1857 participants from the combined Rush datasets. Of these, 989 were designated as non-progressors–cognitively unimpaired participants who were not diagnosed with MCI or AD dementia during the follow-up period. In the PLS regression, correction for normal aging and practice effects was done using data from participants within a 7-year buffer from diagnosis. The “normal” group used for the age correction required a follow-up 7-year time period with no diagnosis and included 590 participants.

**Fig. 1:**
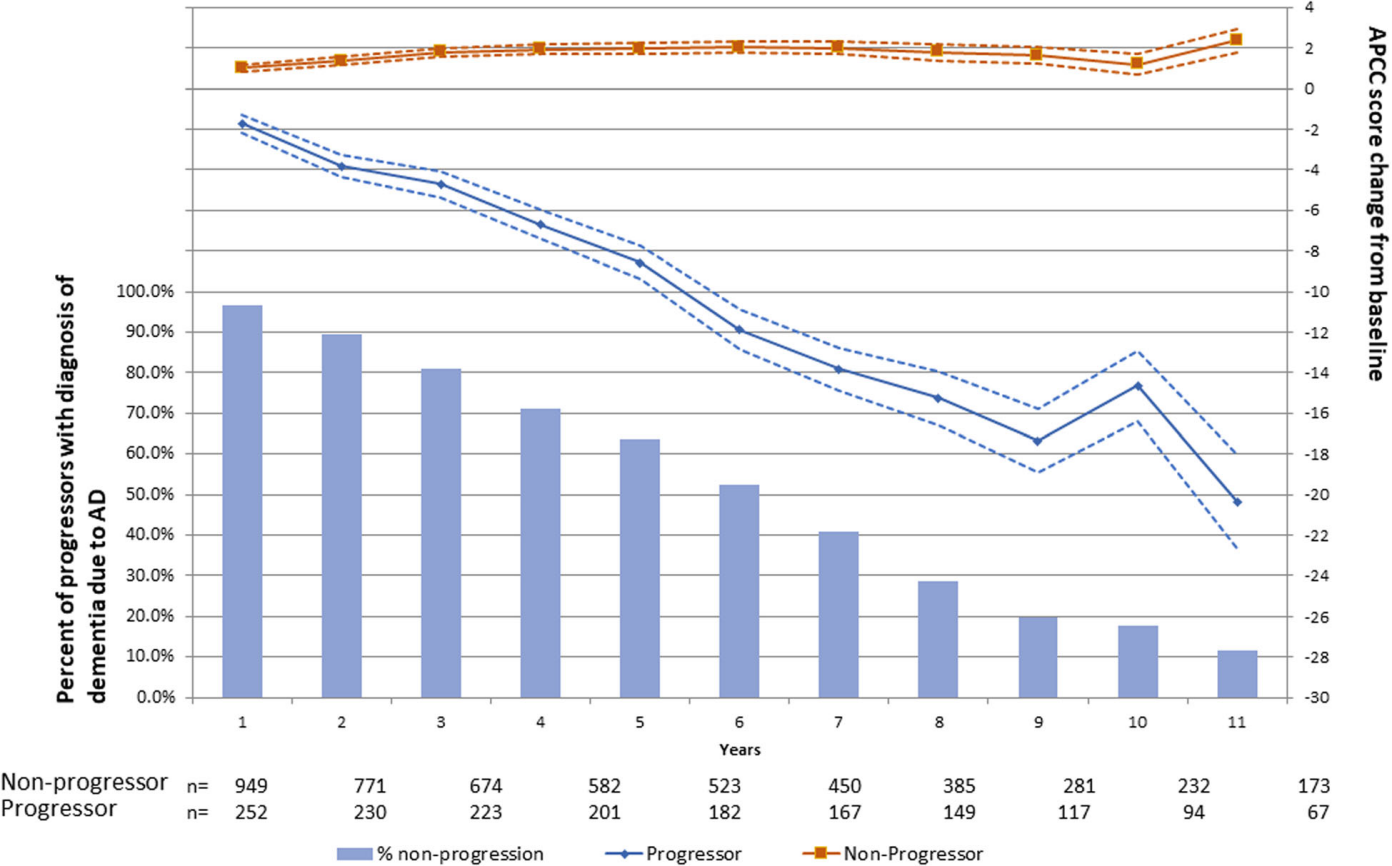
Decline in APCC over 10 years in progressors to MCI or AD versus non-progressors among the Rush Cohorts. Note: solid lines = means; dotted lines = standard errors; at baseline, non-progressors and progressors have a mean (standard deviation) APCC score of 65.54 (7.102) and 60.48 (7.539), respectively. Data beyond year 11 is not shown due to low sample size at later time-points. AD, Alzheimer’s disease; APCC, Alzheimer’s Prevention Initiative Preclinical Cognitive Composite

Of the 1857 participants, 868 were classified as progressors because they were diagnosed with either MCI or AD dementia during the follow-up period. The sample sizes for each group at each visit post-baseline are given along the *x*-axis in Fig. 1. This was a mixed population of people close to diagnosis and years away from diagnosis.

Participant demographics are shown in Table 2. The percentage of females was 70.7% in the progressor group and 75.8% in the non-progressor group. The average age was 77.5 years in the progressors and 74.5 years in the non-progressors. The majority of participants in both groups were White (82.7% progressors, 77.6% non-progressors).

**Table 2:** Baseline characteristics of progressors vs. non-progressors

Characteristic	Non-progressors (***N*** = 989)	Progressors (***N*** = 868)	Overall (***N*** = 1857)	***p*** value
Age				
Mean (SD)	74.5 (7.4)	77.5 (6.8)	75.9 (7.3)	< 0.0001
Gender				
Male	24.2%	29.3%	26.5%	< 0.05
Female	75.8%	70.7%	73.5%	
Race				
Native American, Indian	0.3%	0.1%	0.2%	< 0.05*
Black, Negro, African-American	21.9%	16.6%	19.4%	
White	77.6%	82.7%	80.0%	
Asian or Pacific Island	0.1%	0.5%	0.3%	
Missing	0.1%	0.1%	0.1%	
Education (years)				
Mean (SD)	15.9 (3.8)	16.3 (3.7)	16.1 (3.8)	< 0.05
*APOE*4 carrier status				
Carrier	17.0%	23.3%	19.9%	< .0001
Non-carrier	62.0%	69.9%	65.7%	
Missing	21.0%	6.8%	14.4%	
Baseline MMSE				
Mean (SD)	28.9 (1.3)	28.3 (1.7)	28.6 (1.6)	< 0.0001
Baseline APCC				
Mean (SD)	65.5 (7.1)	61.6 (7.1)	63.7 (7.4)	< 0.0001

*p value for race is White vs. other

### Step 1—PLS model results across time periods

The full list of items that were included in the PLS model is shown in Table 1. Items that were selected by any of the PLS analysis models are shown in Tables 3 and 4. Items that showed consistency across both stages of disease included 7 items: Symbol Digit Modalities, Digit Ordering, MMSE Orientation to Time, Logical Memory Delayed, Word List Memory Delayed, Judgment of Line Orientation, and Raven’s Progressive Matrices (subset of 9 items from A and B) (Combination 1) (Table 3).

**Table 3:** Three steps of PLS methodology

	Cognitive domain			
	Perceptual speed/working memory	Orientation	Episodic memory	Visual spatial
**Step 1:** Original PLS Combination (Combination 1)	1) Symbol Digit Modalities (Perceptual Speed) 2) Digit Ordering (Working Memory)	1) MMSE Orientation to Time	1) Logical Memory Delayed 2) Word List Memory Delayed	1) Judgment of Line Orientation 2) Raven’s Progressive Matrices (subset of 9 items from A and B)
**Step 2:** Added MMSE Orientation to Place (Combination 2)	1) Symbol Digit Modalities (Perceptual Speed) 2) Digit Ordering (Working Memory)	1) MMSE Orientation to Time 2) MMSE Orientation to Place	1) Logical Memory Delayed 2) Word List Memory Delayed	1) Judgment of Line Orientation 2) Raven’s Progressive Matrices (subset of 9 items from A and B)
**Step 3:** Removed Digit Ordering (Combination 3)	1) Symbol Digit Modalities (Perceptual Speed)	1) MMSE Orientation to Time 2) MMSE Orientation to Place	1) Logical Memory Delayed 2) Word List Memory Delayed	1) Judgment of Line Orientation 2) Raven’s Progressive Matrices (subset of 9 items from A and B)

**Table 4:** MSDRs for step-by-step iterations from the − 10 to 0 composite applied to different intervals

Composite	− 10 to 0	− 11 to − 1	− 5 to − 1
**Combination 1**: Symbol Digit Modalities Digit Ordering MMSE Orientation to Time Logical Memory Delayed Word List Memory Delayed Judgment of Line Orientation Raven’s Progressive Matrices (subset of 9 items from A and B)	− 1.06	− 0.5331	− 0.4047
**Combination 2**: Symbol Digit Modalities Digit Ordering MMSE Orientation to Time MMSE Orientation to Place Logical Memory Delayed Word List Memory Delayed Judgment of Line Orientation Raven’s Progressive Matrices (subset of 9 items from A and B)	−1.09	− 0.5460	− 0.4172
**Combination 3 (APCC)**: Symbol Digit Modalities MMSE Orientation to Time MMSE Orientation to Place Logical Memory Delayed Word List Memory Delayed Judgment of Line Orientation Raven’s Progressive Matrices (subset of 9 items from A and B)	− 1.10	− 0.5386	− 0.4124

*MMSE* Mini-Mental State Examination, *MSDR* mean-to-standard deviation ratio

### Step 2—results incorporating theoretical considerations

This 7-item combination was then assessed for domain representation. The domains of Perceptual Speed/Working Memory, Episodic Memory, and Visual Spatial each had two items included in the combination. The domain of orientation had only one item, MMSE Orientation to Time, so we tested a combination including MMSE Orientation to Place as a potential second item within this domain. Running the PLS regression with MMSE Orientation to Place added results in improved MSDRs across the three time periods (Combination 2) (Table 4).

### Step 3—MSDRs excluding each individual item one at a time

Based on the MSDRs in step 2, we identified an 8-item combination to move forward with for step 3 of the scale selection process. In step 3, we compared the MSDRs from our selected combination (Combination 2) with each combination of 7 items, excluding one at a time across the three time periods. Results from the PLS model within each time frame prior to a diagnosis are shown in Table 4. The MSDR for the − 10 to 0 time period (which includes the year of diagnosis) is improved with the removal of the Digit Ordering item (Combination 3) as shown in Table 4.

### Step 4—final composite with weights

Table 5 shows the final APCC score after calculating the weights for the raw item scores (step 4). Although the domain of Perceptual Speed/Working Memory only has one item (Symbol Digit Modalities) due to removal of Digit Ordering from the combination, no other sensitive item was available for this domain. Interestingly, the optimized weighting for Symbol Digit Modalities from the PLS regression was approximately twice as large as the other weights resulting in approximately equal weighting (25%) of all 4 domains even with only one item in this domain.

**Table 5:** Composite weights of the Alzheimer’s Prevention Initiative Preclinical Cognitive Composite (APCC)

Domain	Test item	Weight	Maximum possible	Weighted maximum
Perceptual Speed/Working Memory	Symbol Digit Modalities^‡^	0.26	110	28.6
Orientation	MMSE Orientation to Time^‡^	2.24	5	11.2
	MMSE Orientation to Place	2.14	5	10.7
Episodic Memory	Logical Memory Delayed^‡^	0.53	25	13.2
	Word List Memory Delayed	1.36	10	13.6
Visual Spatial	Judgment of Line Orientation	0.68	15	10.2
	Raven’s Progressive Matrices (subset of 9 items from A and B)^‡^	1.39	9	12.5*
	Total Score (sum of weighted maximums)			100

*MMSE* Mini-Mental State Examination

^‡^Item included in original, late-onset AD composite cognitive test score (this composite consisted of Category Fluency [fruits and vegetables], Boston Naming Test [15 items], Logical Memory Delayed Recall, East Boston Naming Test Immediate Recall, Raven’s Progressive Matrices Subset, Symbol Digit Modalities and MMSE Orientation to Time [12])

*Rounded to the nearest 0.1

### Final selected APCC

The PLS regression was run for the three combinations across the three time periods. Combination 3 yielded the highest MSDR over − 10 to 0 time period (− 1.10) among the three combinations, indicating the greatest sensitivity to detect clinical change over time. The APCC is calculated using the formula: APCC test score = 0.26*Symbol Digit Modalities + 2.24*MMSE Orientation to Time + 2.14*MMSE Orientation to Place + 0.53* Logical Memory Delayed Recall + 1.36* Word List-Delayed Recall + 0.68* Judgment of Line Orientation + 1.39* Raven’s Progressive Matrices (subset of 9 items from A and B) (Table 5).

### Performance of the APCC in the rush cohorts

The APCC was derived and optimized using a PLS regression to be able to detect cognitive decline associated with subsequent progression to dementia. Figure 1 illustrates the expected decline combining data across multiple baseline visits from participants who are at differing years prior to a diagnosis. This method of summarization illustrates what might be expected in a clinical trial population over time. It is important to note that these decline estimates may be exaggerated due to overfitting since they are calculated in the same dataset used to derive the optimal composite score.

## Discussion

Composite cognitive tests are being used increasingly as primary endpoints in AD trials, particularly preclinical trials since traditional outcome measures are not sensitive to tracking preclinical cognitive decline. We reported previously an empirically derived composite cognitive test score sensitive to tracking decline up to 5 years prior to the clinical diagnosis of MCI or dementia [12]. The current study expands upon this previous work in preparation for the API Generation Program trials [14], identifying a new composite cognitive test score sensitive to tracking preclinical AD decline up to 11 years prior to diagnosis. This refined composite cognitive test score has several tests in common with the original, late-onset AD composite cognitive test score [12] as well as the ADCS Preclinical Alzheimer Cognitive Composite (PACC) [10], but does include tests not identified previously. This test score derived in the current study, referred to as the APCC, was a primary outcome in the recently discontinued API Generation Program trials [14], with a slight modification. Although the original derivation was performed with the assessment versions available in the Rush dataset, the Generation Program replaced some of the subtests used in the Rush cohorts (CERAD Word List – Delayed Recall, Logical Memory – Delayed Recall, Symbol Digit Modalities, Judgment of Line Orientation) with nearly identical subtests from the Repeatable Battery for the Assessment of Neuropsychological Status (RBANS) (Delayed Memory – List Recall, Delayed Memory – Story Recall, Coding, Line Orientation) [30]. The respective RBANS assessments are nearly identical to the versions used in the Rush cohorts while providing alternate test forms and linguistically validated translations, which are essential in a global clinical trial. Further information regarding the analyses to support substituting the items from the Rush battery with RBANS assessments will be provided in a future publication.

For the present study, we used data from three large cohort studies that followed participants over many years. The dataset includes data from participants who progressed to the clinical stages of AD dementia as well as from participants who remained cognitively unimpaired during the time of follow-up, allowing us to correct for aging and practice effects. Importantly, this variability in progression is reflective of what might be observed in a preclinical trial population since not everyone who enrolls in a trial will progress to the clinical stages of AD, nor will they progress at the same time or rate. Our analytical approach afforded us the opportunity to examine data from several different time periods, with the goal of ensuring the year in which a diagnosis of MCI or AD dementia did not determine the selection of tests in the composite. Rather, we wanted to identify a composite sensitive to tracking preclinical AD cognitive decline over a range of years prior to diagnosis. This is necessary since not all participants enrolled in a preclinical trial will progress to the clinical stages of AD. Moreover, we selected the combination of items most sensitive to changes in disease progression with the assumption that a disease-modifying treatment will proportionally affect each item and the overall change in the combination [13]. The resulting composite is well-suited for use as a primary endpoint in a preclinical AD trial.

Similar to our previously reported composites for tracking preclinical cognitive decline associated with sporadic, late-onset AD (LOAD), or autosomal-dominant AD (ADAD) [11, 12], we used an empirical strategy supported by theoretical understanding of the cognitive changes that occur during the preclinical stage of the disease to inform the development of the APCC. Specifically, we used our knowledge about the cognitive changes occurring during the preclinical stages of the disease to influence analyses beginning at step 2. Although several composites scores perform similarly to one another (i.e., had nearly identical MSDRs), not all were supported by theoretical understanding of the disease progression. Since the MSDRs were nearly identical, we moved to a combined, empirically derived, theoretically supported approach. Interestingly, there were considerable similarities in the cognitive domains and tests of the APCC and those identified in our prior work [11, 12] as composites developed by other groups [10]. This overlap is noteworthy given that the APCC used a different methodological approach and sensitivity measure (PLS and MSDR) and included different test battery items than the other composites, in addition to differences in the populations. These similarities suggest that there is likely not one “best” composite for tracking cognitive decline in preclinical AD. Rather, it is most important for a composite to include tests measuring the cognitive domains that decline during this stage of the disease (i.e., the specific episodic word list test does not matter as much as including a word list assessment that measures appropriately this aspect of episodic memory). Taken together, the findings from this study support the APCC as an effective measure of preclinical cognitive decline up to 11 years before MCI or AD dementia onset.

The current study used a PLS regression model which allowed us to compare the longitudinal progression between a group of participants who progressed to the clinical stages of the disease during an 11-year time period compared to a group of participants who did not progress. This is in contrast to our prior work which relied on data from discrete time periods (2 and 5 years before clinical progression) to identify the composite with the highest MSDR [12]. In addition, the current study used PLS models to estimate the optimal weighting for each individual test item or component of the composite, rather than first identifying all items (i.e., the composite) and then applying weights. This is important since the individual test items included in the composite are already implicitly weighted due to their unique scale properties. Even if explicit weights are not applied, the method of standardization implicitly weights items. The PLS model allowed us to determine whether the scales’ implicit weighting was appropriate, or whether alternate weighting should be considered when the items were combined into a single composite score, thereby emphasizing the cognitive domains that are declining the most during this stage of the disease. For the APCC, although the cognitive domains are weighted almost equally, the individual tests are not. The APCC, with weighting provided from the PLS model, has a higher MSDR than the composite derived by our prior work [12], suggesting that optimal weighting does generate a more sensitive composite [13]. Lastly, the PLS model allows for the identification of the combination of test items that are aligned with the progressive aspects of the disease rather than optimizing separation between cross-sectional groups (e.g., progressors compared to non-progressors, or biomarker-defined “at risk” compared to “not at risk”).

There are some limitations to the present study. Participants enrolled in the Rush cohort studies may not be reflective of the general population, though are likely to be similar to those enrolled in an AD prevention trial. As with our prior work, development of the APCC was constrained by the overall cognitive test battery used in the observational studies that provided the data to us. There may be “better” or more optimal composites for preclinical AD. We do note, however, that the APCC consists of tests also included in other cognitive composites [10–12]. Future efforts should include direct comparison of methodology to derive composites in addition to comparisons of the sensitivity of the other composites. Until longitudinal, observational cohort studies include novel cognitive assessments in addition to traditional paper and pencil assessments used to date, it may be difficult for the field to identify more sensitive composites, though efforts should continue to refine existing composites [31]. Similarly, as we identified with the MMSE, it may be that only specific items of a test are ideally suited to include in a composite. As such, it is important for these same observational cohort studies to consider including item level data and related information in the database for further refinement of composite scores [32]. Moreover, modifications may be needed to deploy the composite in a global preclinical trial. The observational study that provided the data to develop the APCC used cognitive assessments, which did not have multiple, psychometrically equivalent, alternate test forms nor underwent linguistic validation. Alternate test forms are important for trials since the tests are administered to participants on a regular basis (e.g., every 6 months) and using the same test repeatedly would likely introduce practice and learning effects. Similarly, linguistic validation is important to ensure that the translated assessment has equivalent construct value and is conceptually equivalent across multiple languages and cultures. In order to overcome these issues and to use the APCC in the API Generation Program, a decision was made to substitute four tests used in the Rush studies with nearly identical tests from the RBANS. Two components, however, did not have alternate test forms: the MMSE (Orientation to Time, Orientation to Place), and the Raven’s Progressive Matrices (subset of 9 items from A and B). Future work could focus on further refining the composite to ensure all tests have alternate test versions, are relevant across different cultures, and are not prone to subjective scoring. Similarly, substitution of some items from the RBANS may be a limitation since we did not have longitudinal data to ensure a similar MSDR to the version developed using tests from the Rush studies. In addition, the APCC is likely only suited for use as an endpoint in a preclinical AD trial since it was optimized to track preclinical cognitive decline. A different composite endpoint would be needed for trials enrolling patients with MCI, prodromal AD, or dementia due to AD [33, 34]. It is important to note that while the APCC is sensitive to tracking cognitive decline in preclinical AD, the clinical meaningfulness of change in the APCC over time remains unknown. This is an issue faced by preclinical AD studies using novel composite cognitive endpoints [35]. The Insights to Model Alzheimer’s Progression in Real Life (iMAP) study was intended to be a 5-year, multinational, prospective, longitudinal, non-interventional cohort study collecting data across the spectrum of AD to establish the clinical meaningfulness of the APCC and RBANS by examining their ability to predict clinically meaningful outcomes such as diagnosis of MCI or dementia due to AD, change in Clinical Dementia Rating (CDR) – Global Score [36]. The iMAP study was recently stopped in the context of the planned early termination of the Generation Program, but elements of it may be relevant to future work conducted by us or others. In the absence of iMAP, a time-based interpretation is one approach to how much change on the APCC (or another composite) is clinically relevant. For example, if treatment is associated with extending participants’ cognitive performance for 50% longer compared to placebo.

## Conclusions

The APCC is an empirically derived composite cognitive test score that is sensitive to preclinical AD decline up to 11 years prior to diagnosis of the clinical stages of AD. We selected the combination most sensitive to changes in disease progression with the assumption that a disease-modifying treatment will proportionally affect all progression. The items comprising the APCC are supported by theoretical understanding of cognitive decline that occurs during preclinical AD. The components of the APCC were modified slightly, substituting four tests used in the Rush studies with nearly identical tests from the RBANS, so that the APCC could be used in a global clinical trial. Future efforts will focus on establishing the clinical meaningfulness of the APCC as well as direct comparisons of the sensitivity of the APCC to other cognitive composites.

## Data Availability

The data that support the findings of this study are available from Rush Alzheimer’s Disease Center (RADC) but restrictions apply to the availability of these data, which were used under license for the current study, and so are not publicly available. Requests for data should be directed to the RADC https://www.radc.rush.edu/.

## Abbreviations

AD: Alzheimer’s disease
ADAD: Autosomal-dominant AD
APCC: Alzheimer’s Prevention Initiative Preclinical Composite Cognitive test
API: Alzheimer’s Prevention Initiative
CDR: Clinical Dementia Rating
CERAD: Consortium to Establish a Registry for Alzheimer’s Disease
iMAP: The Insights to Model Alzheimer’s Progression in Real Life
LOAD: Late-onset AD
MAP: Memory and Aging Project
MARS: Minority Aging Research Study
MCI: Mild cognitive impairment
MMSE: Mini-Mental State Examination
MSDR: Mean-to-standard deviation ratio
PLS: Partial least squares
RADC: Rush Alzheimer’s Disease Center
RBANS: Repeatable Battery for the Assessment of Neuropsychological Status
ROS: Religious Orders Study
VIP: Variable Importance for Projection
